# A Perspective on the Mediterranean Diet: A Shift Towards Adaptability

**DOI:** 10.1002/alz70860_104528

**Published:** 2025-12-23

**Authors:** Sanjana Punyamurthula, Mary Catherine Stovall, Addison Stone, Michele Longo, Rebecca J Solch‐Ottaiano

**Affiliations:** ^1^ Tulane University, New Orleans, LA, USA

## Abstract

**Background:**

The MeDi emphasizes the consumption of foods high in fiber, nuts, and healthy fats such as olive oil. Its neuroprotective benefits are often attributed to its high antioxidant and polyphenol content. However, it remains unclear whether these components are solely responsible for the health benefits, or if other factors, such as practices and nutrient profiles present in diets beyond the Mediterranean region, also contribute. As the MeDi is one of the most physician‐recommended diets, it is crucial that these recommendations are mindful of cultural dietary patterns to encourage better adherence.

**Objective:**

There is a growing body of evidence supporting the link between brain health and nutrition, suggesting that dietary interventions can help reduce the risk of Alzheimer's disease. However, nutritional recommendations must be accessible and adaptable to diverse populations. This literature review examines the origins of the MeDi and its cultural accessibility. We explore how MeDi‐based recommendations have been adapted for individuals with varying dietary patterns and the challenges associated with these adaptations.

**Method:**

A literature review was conducted from October 2023 – September 2024 using the databases CINAHL, PubMed, and Web of Science. Examples of terms searched include “Mediterranean diet” AND (“cultural adaptation” OR “cultural sensitivity” OR “cultural diversity”), “Mediterranean diet” AND “patient education” OR “communication”.

**Results:**

Current MeDi scoring tools emphasize foods common in Spain, Italy, and Greece, from where the diet originates, making them less applicable to the culinary traditions of the 19 remaining countries in the Mediterranean region. Including regional foods improves dietary assessment accuracy. Interventions that incorporate traditional recipes into dietary plans enhance satisfaction and compliance, further supporting the importance of culturally inclusive dietary approaches. When adapting the MeDi, recommendations should remain evidence‐based, particularly the inclusion of extra virgin olive oil and nuts.

**Conclusion:**

We suggest using a more accurate term for the MeDi, as the nomenclature may inadvertently exclude regions within the Mediterranean not part of its original framework. These exclusions can create barriers to adherence. A broader framework is needed to account for a diverse population, ensuring that the neuroprotective benefits of the MeDi are accessible to individuals from all backgrounds.

